# Supplementary material for: The interactome of intact mitochondria by cross-linking mass spectrometry provides evidence for coexisting respiratory supercomplexes
Source: Mol Cell Proteomics. 2017 Dec 8;17(2):216–32. doi: 10.1074/mcp.RA117.000470 (PMC5795388; doi:10.1074/mcp.RA117.000470)
Supplement: Supplemental Data [file supp_RA117.000470_133922_0_supp_23978_fzffwf.zip › spectra_annotation/mito_DR_spectra_annotation/201-1-2-1-22-1.pdf]

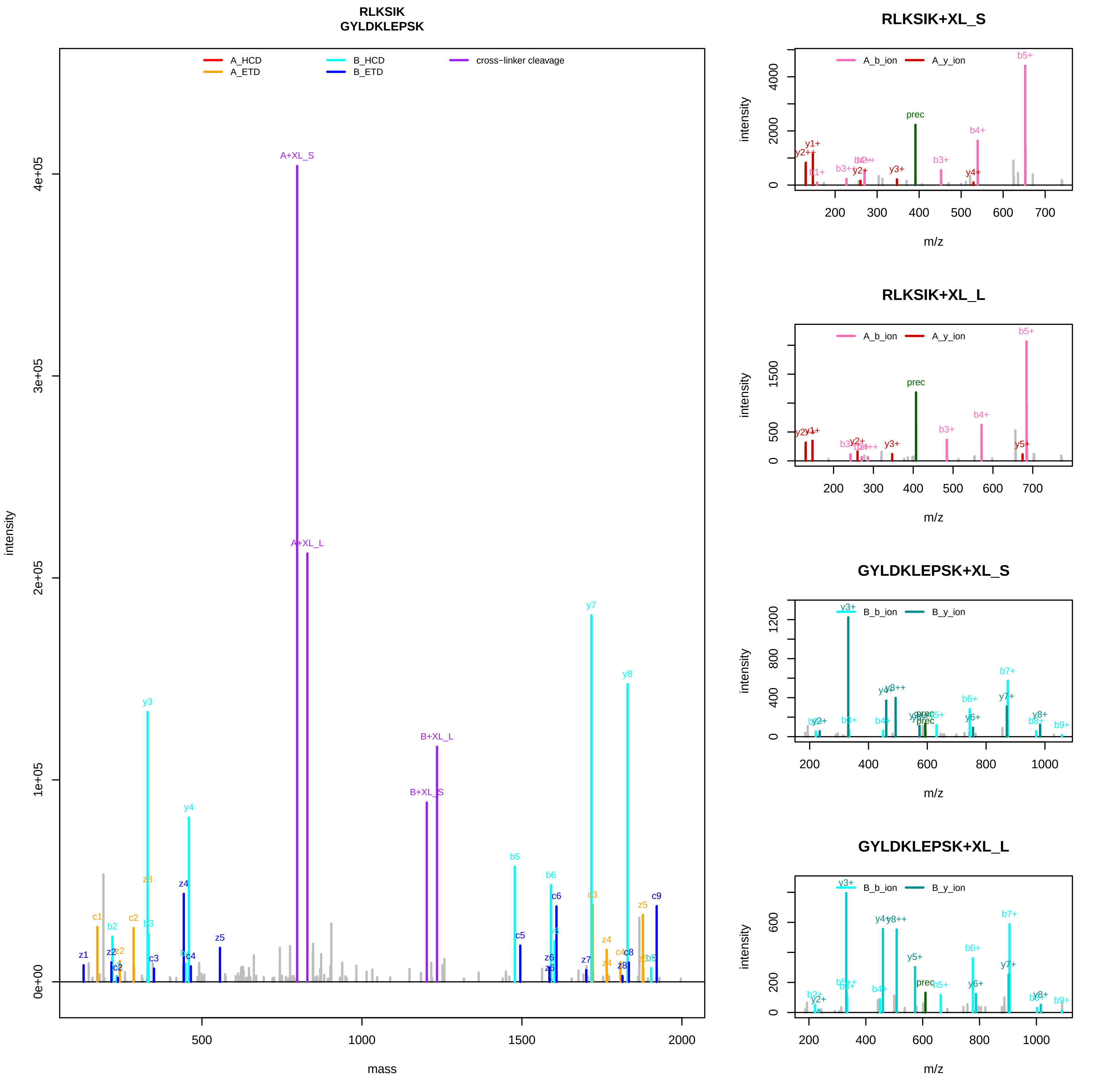
RLKSIK
GYLDKLEPSK
A_HCD
A_ETD
B_HCD
B_ETD
cross−linker cleavage
intensity
mass
0e+00
1e+05
2e+05
3e+05
4e+05
500
1000
1500
2000
A+XL_S
A+XL_L
B+XL_L
B+XL_S
y3
y4
y7
y8
b5
b6
z1
z2
z4
z5
z7
z8
c1
c2
c3
c4
c5
c6
c8
c9
b2
b3
z3
RLKSIK+XL_S
RLKSIK+XL_L
GYLDKLEPSK+XL_S
GYLDKLEPSK+XL_L
A_b_ion
A_y_ion
B_b_ion
B_y_ion
m/z
prec
b5+
b4+
b3+
y1+
y2+
y3+
y4+
y5+
y3+
y4+
y7+
y8+
b6+
b7+
b9+
b8+
y6+
y5+
y8++
0
200
300
400
500
600
700
1000
1500
2000
4000
800
1200
